# Assessing the Economic Burden and Health Care Utilizations of U.S. Veteran Patients with Schizophrenia

**Published:** 2013-10-08

**Authors:** Lin Xie, M. Furaha Kariburyo, Juan Du, Yuexi Wang

**Affiliations:** 1 STATinMED Research, Ann Arbor, MI, USA

**Keywords:** health care utilizations, u.s. veteran, burden of illness, schizophrenia

## Abstract

**Objective:** To examine the economic burden and health care utilizations of schizophrenia in the U.S. veteran population.

**Methods:** A retrospective database analysis was performed using the Veterans Health Administration (VHA) Medical SAS® datasets from October 1, 2008 through September 30, 2012. Patients diagnosed with schizophrenia were identified, and the initial diagnosis date was designated as the index date. A group of patients without schizophrenia of the same age, region, gender and index year were identified and matched by baseline Charlson Comorbidity Index (CCI) score, as the comparison group. Patients in both groups were required to be at least age 18 years and have continuous medical and pharmacy benefits 1 year pre- and 1 year post-index date. One-to-one propensity score matching was used to compare health care costs and utilizations during the follow-up period between the schizophrenia and comparison group patients, adjusted for baseline demographic and clinical characteristics.

**Results:** A total of 171,086 eligible patients were identified for the schizophrenia and control cohorts. After 1:1 matching, a total of 70,045 patients were matched from each cohort with well-balanced baseline characteristics. Patients diagnosed with schizophrenia had significantly higher health care utilization in inpatient (18.12% vs. 2.30%, p<0.01), emergency room (19.67% vs. 6.46%, p<0.01), office (98.32% vs. 53.26%, p<0.01), and outpatient visits (98.53% vs. 54.16%, p<0.01). Higher health care utilizations translated into higher costs for schizophrenic patients including inpatient ($7,228 vs. $613, p<0.01), pharmacy ($1,012 vs. $343, p<0.01), outpatient ($3,998 vs. $1,302, p<0.01), and total costs ($12,238 vs. $2,260, p<0.01) relative to patients in the comparison group.

**Conclusion:** Patients diagnosed with schizophrenia in the U.S. VHA system were associated with a substantial economic burden, compared to their matched controls.

[1]STATinMED Research, Ann Arbor, MI, USA *Corresponding author lxie@statinmed.com

## 1. Introduction

Schizophrenia is a chronic, severe and disabling brain disorder caused by genetics and environmental factors, and is one of the most costly mental illnesses, in terms of economic and social burden.[1] In the absence of clear biological markers, schizophrenia has historically been diagnosed according to signs and symptoms.[2] The symptoms of schizophrenia usually fall into three broad categories: positive symptoms, negative symptoms and cognitive symptoms. Positive symptoms are psychotic behaviors such as hallucinations, delusions and thought and movement disorders. Negative symptoms and cognitive symptoms are difficult to recognize and may include, among other symptoms, lack of pleasure and trouble focusing.[3]

In 2011, schizophrenia prevalence was estimated to be between 0.3% and 0.7% in the United States.[4] Schizophrenia appears early on in male patients with peak ages of onset ranging between 20 to 28 years, compared to age 26 to 32 years in female patients. Male patients are also 1.4 times more affected by schizophrenia than female patients.[5] Schizophrenic patients die 12–15 years before the average population, and this mortality difference has increased in recent decades.[1] People with schizophrenia have a mortality risk that is two to three times that of the general population.

The main purpose of schizophrenia treatment is to control psychotic symptoms and improve patients' societal functioning.[7] Schizophrenia therapies include antipsychotic medications and various psychosocial treatments. Typical antipsychotics have been available since the mid-1950s. New atypical antipsychotics were developed in the 1990s, such as clozapine (Clozaril®), olanzapine (Zyprexa®) and risperidone (Risperdal®), and are thought to improve negative symptoms.[8]

In 2002, overall costs of schizophrenia in the United States were estimated at $62.7 billion, with $22.7 billion excess direct health care costs. The total direct non-health care excess costs, including offset living costs, were estimated at $7.6 billion.[9] From 1991 to 2002, a decrease in hospitalization costs has been accompanied by a substantial increase in outpatient and medication costs, due to atypical antipsychotic drug development.[10] In the Nicholls *et al.* 2010 study, recently diagnosed patients were more likely to have an inpatient admission (22.3% vs. 12.4%), had a greater average hospital length of stay (5.1 days vs. 3.0 days) and more frequent emergency room (ER) utilization, when compared with chronic patients.[11]

A higher prevalence of schizophrenia may be observed in U.S. veterans, as they are exposed to traumatic experiences during military service, and have a high possibility of homelessness. In this study, health care resource utilization and costs were compared between U.S. veteran patients with schizophrenia diagnosis and those without schizophrenia diagnosis.

## 2. Methods

*Data Source*

This was a retrospective cohort study using data from the Veterans Health Administration (VHA) Medical SAS® Datasets, which include medical and pharmacy claims and enrollment information. The study period was from October 1, 2008 through September 30, 2012. Two sets of matching were conducted, one during the sample selection and the other during multivariate analysis.

*Sample Selection*

Patients diagnosed with schizophrenia were identified from October 1, 2009 through September 30, 2011. The index date was defined as the initial schizophrenia diagnosis date for the Schizophrenia Cohort. For the control cohort, the index date was randomly assigned, for patients without a schizophrenia diagnosis, within the same identification period. Patients in both cohorts were required to be at least age 18 on the index date, with continuous health plan enrollment for at least 12 months pre- (baseline period) and 12 months post-index date (follow-up period). Patients without schizophrenia were required to have same age, region, gender and index year as those diangosed with schizophrenia. The cohorts were finally matched based on pre-index Charlson Comorbidity Index (CCI) scores using 1:1 propensity score matching (PSM). The cohort sample sizes were equal.

*Baseline Variables*

Baseline demographic variables included age, age group (18-25, 26-34, 35-54, 55-64, 65+ years), gender and U.S. geographic region (Northeast, Midwest, South, West, Unknown). Average pre-index CCI scores were calculated, and individual comorbid conditions listed in the CCI were: myocardial infarction (MI), congestive heart failure, peripheral vascular disease (PVD), dementia, cerebrovascular disease, chronic obstructive pulmonary disease (COPD), rheumatologic disease, peptic ulcer disease, mild liver disease, hemiplegia or paraplegia, renal disease, diabetes, any malignancy including leukemia and lymphoma, moderate or severe liver disease, metastatic solid tumor, diabetes with chronic complications, acquired immune deficency syndrome (AIDS), hypertension, depression, skin ulcers/cellulitis and warfarin use.

*Outcome Variables*

All outcomes were measured for the 12-month follow-up period.

*Health Care Utilization*

Health care utilization (percentage of patients, number of visits) was computed for inpatient admissions, ambulatory (office, outpatient), outpatient emergency room (ER) and pharmacy visits for the follow-up period.

*Health Care Costs*

Follow-up health care costs were calculated as outpatient ER, outpatient office, inpatient admission and pharmacy costs. Total costs were the sum of inpatient, outpatient and pharmacy costs. Costs were adjusted to 2012 U.S. dollars using the medical care component of the Consumer Price Index (CPI).

*Statistical Analysis*

All study variables, including baseline and outcome measures, were analyzed descriptively. Bivariate comparisons of baseline characteristics and outcome measures, stratified by schizophrenia and no schizophrenia cohorts were conducted. Percentages and counts were provided for dichotomous and polychotomous variables. Means and standard deviations were provided for continuous variables. Student t-tests were used for the means of continuous variables. For dichotomous variables, Chi-square tests were used to evaluate the statistical significance of differences in categorical variables. P-values were also provided. To adjust for baseline differences in demographic and clinical characteristics between the cohorts, risk adjustment was performed using 1:1 propensity score matching (PSM) to compare all follow-up health care costs and utilization between the cohorts. Variables incorporated in the matching were: age, gender, geographic region and all baseline individual comorbid conditions.

PSM uses observed characteristics to create a quasi-experimental setting, where the treatment and control group are equal (or matched) based on all observable covariates. Two closely-matched study cohorts were created after matching, and follow-up health care utilization and health care costs were considered to be the true effect of the disease. Propensity scores are estimated by unconditional logistic regression analyses. Each patient in the Schizophrenia Cohort was matched with one patient in the Control Cohort, if their propensity scores were within ±0.0001 units of each other.

## 3. Results

After applying all inclusion and exclusion criteria, a total of 171,086 patients were identified for analysis, with 85,543 patients in each cohort (Figure 1).

**Figure 1. Patient Selection**

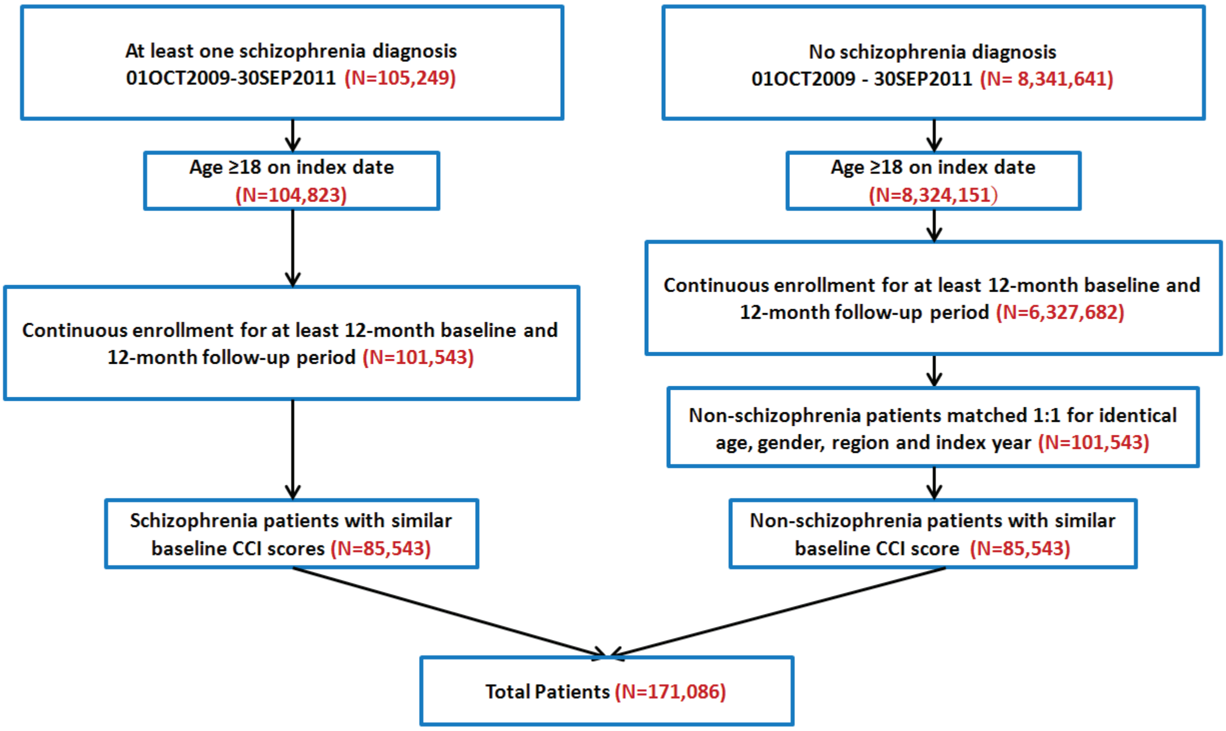

*Demographic and Clinical Characteristics*

Schizophrenic patients were younger on average (56.38 vs. 57.40 years, p<0.0001), compared to control patients. Significant differences were found in age groups 35-54 (34.76% vs. 30.92%, p<0.0001), 55-64 (42.29% vs. 42.97%, p=0.0049) and 65+ (17.95% vs. 21.22%, p<0.0001) (Table 1).

**Table 1. Demographic and Clinical Characteristics of Patients with and without Schizophrenia**

| | | Schizophrenia Cohort (N=85,543) | | Control Cohort (N=85,543) | | | |
|---|---|---|---|---|---|---|---|
| **Baseline Demographic Conditions** | | **N/Mean** | **%/SD** | **N/Mean** | **%/SD** | **p-value** | **Effect Size** |
| **Age** | | 56.38 | 11.60 | 57.40 | 11.93 | <0.0001 | 8.70 |
| | **18-25** | 613 | 0.72% | 651 | 0.76% | 0.2834 | 0.52 |
| | **26-34** | 3,665 | 4.28% | 3,529 | 4.13% | 0.1014 | 0.79 |
| | **35-54** | 29,731 | 34.76% | 26,454 | 30.92% | <0.0001 | 8.16 |
| | **55-64** | 36,180 | 42.29% | 36,755 | 42.97% | 0.0049 | 1.36 |
| | **65+** | 15,354 | 17.95% | 18,154 | 21.22% | <0.0001 | 8.25 |
| **Gender** | | | | | | | |
| | **Male** | 79,715 | 92.88% | 79,449 | 93.19% | 0.0115 | 1.22 |
| | **Female** | 5,828 | 7.12% | 6,094 | 6.81% | 0.0115 | 1.22 |
| **Region** | | | | | | | |
| | **Northeast** | 14,601 | 17.07% | 13,593 | 15.89% | <0.0001 | 3.18 |
| | **Midwest** | 17,868 | 20.89% | 18,198 | 21.27% | 0.0505 | 0.95 |
| | **South** | 28,574 | 33.40% | 29,943 | 35.00% | <0.0001 | 3.37 |
| | **West** | 17,020 | 19.90% | 16,137 | 18.86% | <0.0001 | 2.61 |
| | **Other Region** | 7,480 | 8.74% | 7,672 | 8.97% | 0.1023 | 0.79 |
| **Baseline Comorbid Conditions** | | | | | | | |
| | **Charlson Comorbidity Index score** | 1.59 | 1.88 | 1.59 | 1.88 | 1 | 0.00 |
| | **Myocardial infarction** | 1,089 | 1.27% | 1,415 | 1.65% | <0.0001 | 3.17 |
| | **Congestive heart failure** | 2,281 | 2.67% | 2,266 | 2.65% | 0.8216 | 0.11 |
| | **Peripheral vascular disease** | 2,294 | 2.68% | 3,002 | 3.51% | <0.0001 | 4.78 |
| | **Dementia** | 626 | 0.73% | 205 | 0.24% | <0.0001 | 7.08 |
| | **Cerebrovascular disease** | 3,090 | 3.61% | 3,441 | 4.02% | <0.0001 | 2.14 |
| | **Chronic pulmonary disease** | 13,372 | 15.63% | 10,578 | 12.37% | <0.0001 | 9.42 |
| | **Rheumatologic/Connective tissue disease** | 384 | 0.45% | 799 | 0.93% | <0.0001 | 5.86 |
| | **Peptic Ulcer disease** | 823 | 0.96% | 821 | 0.96% | 0.9605 | 0.02 |
| | **Mild liver disease** | 839 | 0.98% | 820 | 0.96% | 0.6392 | 0.23 |
| | **Hemiplegia or paraplegia** | 288 | 0.34% | 335 | 0.39% | 0.0592 | 0.91 |
| | **Moderate or severe renal disease** | 3,062 | 3.58% | 3,059 | 3.58% | 0.9689 | 0.02 |
| | **Diabetes** | 18,576 | 21.72% | 17,705 | 20.70% | <0.0001 | 2.49 |
| | **Tumor (Other Malignancy)** | 3,939 | 4.60% | 5,049 | 5.90% | <0.0001 | 5.82 |
| | **Moderate or severe liver disease** | 284 | 0.33% | 292 | 0.34% | 0.7385 | 0.16 |
| | **Metastatic solid tumor** | 253 | 0.30% | 256 | 0.30% | 0.8941 | 0.06 |
| | **Diabetes + complications** | 3,392 | 3.97% | 4,178 | 4.88% | <0.0001 | 4.47 |
| | **AIDS** | 600 | 0.70% | 580 | 0.68% | 0.5591 | 0.28 |
| | **Hypertension** | 37,475 | 43.81% | 40,936 | 47.85% | <0.0001 | 8.13 |
| | **Depression** | 18,973 | 22.18% | 15,948 | 18.64% | <0.0001 | 8.78 |
| | **Skin ulcers/cellulitis** | 4,300 | 5.03% | 3,186 | 3.72% | <0.0001 | 6.37 |
| **Baseline warfarin use** | | 512 | 0.60% | 824 | 0.96% | <0.0001 | 4.14 |

SD=standard deviation

Nearly 92% of the identified patients were male, and more than 33% of the participants resided in the South U.S. region. Schizophrenic patients were more likely to reside in the Northeast (17.07% vs. 15.89%, p<0.0001) and West U.S. regions (19.90% vs. 18.86%, p<0.0001), but less likely to reside in the South (33.40% vs. 35.00%, p<0.0001), compared to non-schizophrenic patients.

The matched baseline CCI score level for both cohorts was 1.59. However, significant differences between the two cohorts were observed for baseline individual comorbidities. Non-schizophrenic patients were more often diagnosed with MI (1.65% vs. 1.27%, p<0.0001), hypertension (47.85% vs. 43.81%, p<0.0001), PVD (3.51% vs. 2.68%, p<0.0001), cerebrovascular disease (4.02% vs. 3.61, p<0.0001), rheumatologic or connective tissues disease (0.93% vs. 0.45%, p<0.0001) and tumor (5.90% vs. 4.60%, p<0.0001). Schizophrenic patients were more likely to be diagnosed with dementia (0.73% vs. 0.24%, p<0.0001),

COPD (15.63% vs. 12.37%), diabetes (21.72% vs. 20.70%), depression (22.18% vs. 18.64%) and skin ulcers (5.03% vs. 3.72%). Non-schizophrenic patients were more likely to be prescribed warfarin during the baseline period (0.96% vs. 0.60%, p<0.0001). (Table 1)

*Post-index Descriptive Outcomes*

During the follow-up period, schizophrenic patients had more health care utilizations, including inpatient admissions (19.19% vs. 2.56%, p<0.0001) and outpatient ER (20.52% vs. 6.53%, p<0.0001), outpatient office (98.22% vs. 53.20%, p<0.0001), any outpatient (98.42% vs. 54.12%) and pharmacy visits (90.04% vs. 57.72%, p<0.0001), resulting in significantly higher average health care costs in all subcategories, including inpatient ($7,857 vs. $770, p<0.0001), outpatient ER ($244 vs. $60, p<0.0001), outpatient office ($3,827 vs. $1,177, p<0.0001), total outpatient ($4,206 vs. $1,340, p<0.0001), pharmacy ($1,053 vs. $362, p<0.0001) and total health care costs ($13,116 vs. $2,471, p<0.0001) compared to non-schizophrenic patients. (Table 2)

**Table 2. Post-Index All-cause Health Care Costs and Utilization for Schizophrenia vs. Control Cohorts**

| | Schizophrenia Cohort (N=85,543) | | Control Cohort (N=85,543) | | | |
|---|---|---|---|---|---|---|
| | N/Mean | %/SD | N/Mean | %/SD | P-value | Effect Size |
| **Follow-up Health Care Utilization** | | | | | | |
| Inpatient admissions | 16,419 | 19.19% | 2,192 | 2.56% | <0.0001 | 55.43 |
| ER visits | 17,551 | 20.52% | 5,583 | 6.53% | <0.0001 | 41.80 |
| Office visits | 84,023 | 98.22% | 45,512 | 53.20% | <0.0001 | 123.35 |
| Outpatient visits | 84,190 | 98.42% | 46,300 | 54.12% | <0.0001 | 121.94 |
| Pharmacy visits | 77,021 | 90.04% | 49,379 | 57.72% | <0.0001 | 79.10 |
| **Follow-up Health Care Costs** | | | | | | |
| Inpatient Costs | $7,857 | $34,301 | $770 | $11,209 | <0.0001 | 27.77 |
| ER Costs | $244 | $814 | $60 | $337 | <0.0001 | 29.55 |
| Office Visit Costs | $3,827 | $6,457 | $1,177 | $3,432 | <0.0001 | 51.26 |
| Outpatient Costs | $4,206 | $6,975 | $1,340 | $4,018 | <0.0001 | 50.36 |
| Pharmacy Costs | $1,053 | $3,204 | $362 | $7,560 | <0.0001 | 11.91 |
| Total Costs (Inpatient+Outpatient+Pharmacy) | $13,116 | $35,967 | $2,471 | $14,919 | <0.0001 | 38.66 |

SD=standard deviation; ER=emergency room

*Post-index-Adjusted Outcomes*

After applying PSM, by controlling baseline demographic and clinical differences, a total of 70,045 schizophrenic patients were matched with 70,045 non-schizophrenic patients. Post-matching results were consistent with descriptive health care cost and utilization patterns, as illustrated in Figure 2.

Among the 70,045 matched pairs, more schizophrenic patients had inpatient admissions (18.12% vs. 2.30%, p<0.0001) and ER (19.67% vs. 6.46%, p<0.0001), physician office (19.67% vs. 6.46%, p<0.0001), outpatient (98.53% vs. 54.16%, p<0.0001) and pharmacy visits (89.85% vs. 56.27%, p<0.0001) during the follow-up period, compared to non-schizophrenic patients (Figure 2).

**Figure 2. Adjusted Health Care Utilization among Schizophrenia and Control Cohorts**

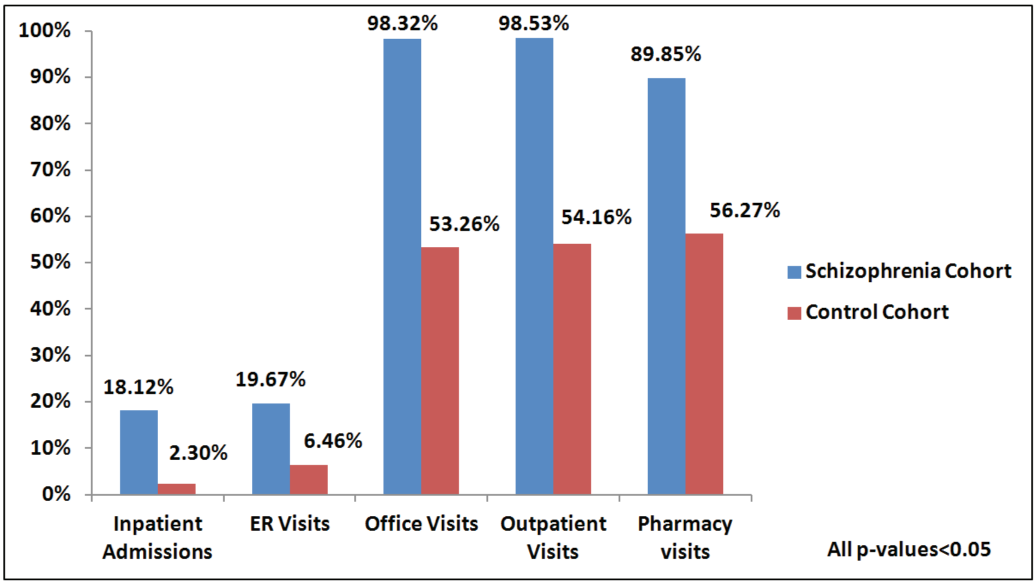

ER=emergency room

Higher health care utilizations resulted in higher health care costs incurred for schizophrenic patients. In fact, they incurred significantly higher inpatient ($7,228 vs. $613, p<0.0001), ER visit ($227 vs. $58, p<0.0001), physician office visit ($3,658, vs. $1,152, p<0.0001), outpatient ($3,998 vs. $1,303, p<0.0001), pharmacy ($1,012 vs. $344, p<0.0001) and total costs ($12,238 vs. $2,260, p<0.0001) compared to non-schizophrenic patients (Figure 3).

**Figure 3. Adjusted Health Care Costs among Schizophrenia and Control Cohorts**

ER=emergency room

## 4. Discussion

Schizophrenia is a chronic mental illness that has substantial economic consequences, particularly on the individual and their families. This retrospective study, performed using the VHA Medical SAS® datasets from October 1, 2009 through September 30, 2012, compared health care costs and utilizations of patients with schizophrenia versus those without the disease.

Risk-adjusted health care costs and utilizations were measure after adjusting for demographic and baseline characteristics. Results showed that the total costs were 5-fold higher for patients diagnosed with schizophrenia compared to those without the disease. Hospitalization costs accounted for the primary difference between the two cohorts, where schizophrenic patients incurred almost 10 times higher inpatient costs than non-schizophrenic patients. In addition, outpatient medical costs, followed by physician office visit costs were three times higher for schizophrenic patients compared to control patients.

This retrospective study focused on schizophrenic patients within the VHA population. According to the National Psychosis Registry, the VHA treated 94,395 schizophrenic patients in the 2003 fiscal year, with costs totaling $1.64 billion. Many of these patients had multiple medical or substance abuse comorbidities. In fiscal year 2002, out of the reported $373.3 million in VHA outpatient psychiatric medication costs, 91% were attributed to depression and schizophrenia medications.[13]

There is also a wide variety of research that has been conducted on schizophrenia burden of illness. Consistent with the findings in the current study, most studies have determined that inpatient costs account for the majority of direct costs for schizophrenia,[4,14-16] driven largely by relapse and re-hospitalization, due to non-compliance with medication, leading to more frequent and severe episodes of psychosis.[17-21] A study conducted in England found similar results, reporting that inpatient length of stay accounted for a significant proportion of schizophrenia-related costs, including approximately 38% of all health, social and institutional care, and roughly 21% of all public sector costs were associated with the condition.[22] Although many factors influence rehospitalization, medication non-adherence is a significant contributor.[16] The current study focused only on the economic burden of schizophrenia, and adherence was not included as one of the studied outcomes. However, Weiden *et al.* estimated in their study that 40% of rehospitalizations costs among persons with schizophrenia were attributable to non-adherence.[19]

Established statistical methods were used to control for biases. However, there were limitations to this study. This study used claims data, and while claims data are extremely valuable for the efficient and effective examination of health care outcomes, treatment patterns and health care resource utilization and costs, they are collected for the purpose of payment, not research. Therefore, certain limitations are associated with the use of claims data.[23] First, certain information is not readily available in claims data that could have an effect on study outcomes, such as clinical and disease-specific parameters. Second, medications filled over-the-counter or provided as samples by the physician are not observed in claims data. Finally, the presence of a claim for a filled prescription does not indicate that the medication was consumed nor that it was taken as prescribed.

Although the VHA dataset contains uniquely integrated data, its beneficiaries are considered a vulnerable population. Research has demonstrated that numerous VHA recipients are older men who are economically disadvantaged and have a high disease burden.[24] Therefore, they may not represent typical schizophrenic patients, which may limit the generalizability or external validity of the study.

## 5. Conclusion

The health care burden of schizophrenia remain a considerable issue in the veteran population, due to higher prevalence for being exposed to traumatic experiences during military service, and the high possibility of homelessness. Veterans' psychiatric and medical conditions affect their functional and occupational skills, rendering them vulnerable to additional stressors, such as higher costs for essential medications.

As a retrospective analysis of claims data from the VHA Medical SAS® datasets, the study findings indicate that schizophrenia is a costly disease resulting in significant costs in excess of those incurred by matched control patients. In fact, schizophrenic patients were associated with higher health care utilizations, leading to a higher health care cost burden, especially for inpatient costs. The current study provides additional cost estimates of schizophrenia, which adds new information to the available literature. By considering each of these costs drivers, health care providers can subsequently tailor schizophrenia interventions to those with the potential to reduce the economic impact of these drivers, thereby improving the health and well-being of schizophrenic patients and their families.

### Acknowledgements

The authors would like to thank Lu Li, MS, of STATinMED Research for her statistical programming in this study.

### Conflict of Interest Declaration

The authors have no conflicts of interest to declare, as this study was not funded.
